# Beyond the pelvis: diagnostic and surgical challenges of thoracic endometriosis syndrome - a retrospective cohort study of 41 patients

**DOI:** 10.3389/fmed.2026.1835516

**Published:** 2026-05-28

**Authors:** Natalia Szczepańska, Przemysław Miłosz, Rafał Pęksa, Aleksandra Ciarka, Marcin Folwarski, Julia Niedzielska, Michał Szymański, Maciej Wilczyński, Tomasz Marjański

**Affiliations:** 1 Faculty of Medicine, Medical University of Gdansk, Gdansk, Poland; 2 Faculty of Medicine, Department of Clinical Pathomorphology, Medical University of Gdansk, Gdansk, Poland; 3 Department of Clinical Nutrition and Dietetics, Faculty of Health Sciences with the Institute of Marine and Tropical Medicine, Medical University of Gdansk, Gdansk, Poland; 4 Faculty of Medicine, Department of Surgical Oncology, Transplant and General Surgery, Medical University of Gdansk, Gdansk, Poland; 5 Faculty of Medicine, Department of Thoracic Surgery, Medical University of Gdansk, Gdansk, Poland

**Keywords:** catamenial pneumothorax, diaphragmatic reconstruction, immunohistochemistry, thoracic endometriosis syndrome, video-assisted thoracoscopic surgery

## Abstract

**Objective:**

This study provides a comprehensive analysis of surgically managed thoracic endometriosis syndrome (TES) patients, primarily assessing whether diaphragmatic reconstruction is associated with lower postoperative recurrence. Secondarily, the study aims to evaluate histopathological findings and temporal changes in management of TES at a tertiary care center.

**Methods:**

This retrospective study of 41 female patients (median age 37) treated surgically for TES between 2012 and 2025 at the Department of Thoracic Surgery, Medical University of Gdansk, Poland. Patients presenting with pneumothorax and/or symptoms of diaphragmatic endometriosis were stratified into Early and Late periods to evaluate changes in surgical management - video-assisted thoracoscopic surgery (VATS), thoracotomy, thoracolaparoscopy, and the implementation of accurate immunohistochemical histopathological assessment.

**Results:**

Complete follow-up was available for 78.0% of patients, however, with differential loss to follow-up between groups (4.5% reconstruction vs. 42.1% no reconstruction; *p* = 0.006) may have biased results, with the direction of bias depending on the outcomes of patients lost to follow-up. Among patients with complete follow-up, intervention-requiring recurrence occurred in 0% of women with reconstruction versus 27.3% without reconstruction (Fisher exact *p* = 0.033). Among patients treated since 2022, diaphragmatic reconstruction was more frequent than in 2012–2021 (71.4% vs. 35.0%, *p* = 0.029). Routine immunohistochemical staining since 2022 improved histopathological confirmation of TES (90% vs. 0%; *p* < 0.001).

**Conclusion:**

In the study, diaphragmatic reconstruction was associated with lower intervention-requiring recurrence, which may indicate a potential benefit of reconstructive techniques in TES surgery. Routine immunohistochemical evaluation (ER, PR, CD10) may be essential for TES diagnosis.

## 1 Introduction

Endometriosis is a chronic disease, characterized by the presence of endometrial-like glands and stroma outside of the uterine cavity (1). Extrapelvic endometriosis is reported in approximately 9–15% of patients (2). Thoracic endometriosis syndrome (TES) represents the most common site of involvement outside the pelvis (3) remains a diagnostic and therapeutic challenge with classification that continues to be debated (4). Although a recently suggested framework subdivides TES into pleural, bronchopulmonary, and diaphragmatic forms (4), these types are frequently closely related. Patients present clinical manifestations resulting from endometrial lesions within the thoracic cavity, such as pneumothorax, hemothorax, hemoptysis associated with menstruation or cyclical and noncyclical chest and/or shoulder pain (5). In many patients, pneumothorax develops secondary to diaphragmatic lesions caused by endometriotic foci, indicating a shared pathophysiological background (2).

Despite its clinical impact, TES is frequently underdiagnosed. Accurate histopathological confirmation of TES is essential but frequently underutilized. Hematoxylin and eosin (H&E) staining is often insufficient for detecting the endometrial foci typically found in thoracic tissue samples. Thus, the immunohistochemical (IHC) staining for estrogen receptor (ER), progesterone receptor (PR), and CD10 significantly increases diagnostic accuracy (6, 7).

Current TES management remains limited by not yet well established standardized surgical strategies and insufficient evidence regarding the role of diaphragmatic reconstruction in recurrence prevention. Furthermore, the underutilization of IHC techniques and the absence of standardized diagnostic and therapeutic pathways mean that management remains diverse. There is a need for more data regarding long-term outcomes to bridge these gaps in clinical practice.

The study aims address these gaps by providing a comprehensive analysis of surgically treated patients with TES, integrating clinical presentation, diagnostic approaches, pathological confirmation, and surgical treatment of TES. Considering the diagnostic complexity of TES, this study emphasizes the necessity of collaboration among thoracic surgeons, gynecologists, and pathologists. The standardization of diagnostic pathways, particularly in the domain of histopathological evaluation, together with choice of hormonal therapy and minimally invasive surgical techniques, holds the potential to enhance long-term outcomes and minimize recurrences.

## 2 Materials and methods

### 2.1 Study design and setting

This is an original article presenting retrospective analysis of 41 patients operated on, between January 2012 and October 2025, in the Department of Thoracic Surgery of Medical University of Gdansk, Poland, capturing the evolution of surgical management for TES. All patients accepted for surgery were enrolled in the study, and the complete analyzed data set was included in the final analysis. Patient recruitment occurred from January 2012 through October 2025. Data collection was completed in December 2025, with the last follow-up visit. This study is reported in accordance with the Strengthening the Reporting of Observational Studies in Epidemiology (STROBE) guidelines for cohort studies. The completed STROBE checklist is provided as Supplementary material.

### 2.2 Participants

Patients were identified from a prospectively maintained institutional database that included all relevant clinical data for individuals who underwent surgical treatment for TES or catamenial pneumothorax during the study period at the Department of Thoracic Surgery, Medical University of Gdansk. Inclusion criteria for the retrospective analysis were symptoms at presentation suggestive of TES, including documented pneumothorax and/or symptoms characteristic of diaphragmatic endometriosis, such as cyclical or noncyclical shoulder pain, arm pain, and recurrent chest pain. These pain symptoms typically occurred in the absence of radiologically evident pneumothorax.

A follow-up concerning recurrence and hormonal treatment was recorded for 32 patients (78.0%) in the study. The follow-up data included recurrence of pneumothorax requiring surgical reintervention or chest tube placement, recurrence of pneumothorax, recurrence of pain and hormonal therapy after and before surgical treatment. The median hospitalization time was 6 days, and the median operative time was 85 min. Follow-up duration ranged from 3 to 156 months (median 36 months). The last follow-up contact was recorded in December 2025. Patients were considered lost to follow-up if they had no documented contact for >12 months. Efforts to minimize loss to follow-up included telephone outreach. The sample size was determined by consecutive enrolment of all eligible patients treated at our institution during the study period (convenience sample). No formal *a priori* sample size calculation was performed.

### 2.3 Data collection and definitions

Clinical data were extracted from electronic medical records. Baseline variables included age in years at the time of index surgery, smoking status (categorized as current or former smoker vs. never smoker), and parity (nulliparous vs. parous). Laterality was categorized as right, left, or bilateral based on operative findings and preoperative imaging. The number of previous pneumothoraces was documented based on episodes prior to index surgery. The study period was separated into Early (surgery performed 2012–2021) and Late (surgery performed 2022–2025) eras to account for temporal changes in surgical pathways. The operative approach was categorized as video-assisted thoracoscopic surgery (VATS), thoracotomy, or combined approach - thoracolaparoscopy. Concurrent procedures included pulmonary wedge resection and/or pleurectomy performed during surgery. It is worth noting that most patients were operated on at a single center, predominantly by one general thoracic surgeon (T.M.), and that some patients underwent more than one surgical procedure. In cases performed via thoracolaparoscopy, the procedures were conducted jointly by a general thoracic surgeon (T.M.) and general surgeons (M.S. and M.W.). The choice of surgical procedure was determined by the operating surgeon based on individual patient characteristics and intraoperative findings. Patients who underwent diaphragmatic reconstruction were identified through operative report review, defined as any procedure involving repair or resection and reconstruction of the diaphragm for porous defects. Use of preoperative and postoperative hormone therapy (HTx) was also documented. The primary outcome was intervention-requiring recurrence, defined as a composite of recurrent pneumothorax requiring chest tube insertion or recurrent symptoms requiring repeat operative procedure. Secondary outcomes included recurrence of pneumothorax, recurrence of pain and any recurrence (pain recurrence, pneumothorax recurrence, or intervention-requiring recurrence). Follow-up data were extracted from all available clinic notes and hospital admissions through the end of the study period. Data quality was assured through automated range and logic checks, with queries resolved through source document verification. Additionally, recorded patients were divided into two groups of females operated on until end of 2021 and patients treated surgically since 2022. This prospective division is based on a retrospective analysis of histopathological results, in which the appropriate use of staining for estrogen receptors (ER), progesterone receptors (PR) and CD10 was observed at the Department of Clinical Pathomorphology, Medical University of Gdansk, since 2022.

The standardized VATS approach included positioning the patient in the lateral decubitus position, utilizing double-lumen intubation with one-lung ventilation, and making incisions as follows: a port in the 7th intercostal space in the anterior axillary line (30° scope), a 3-centimetre utility incision in the 5th intercostal space with a soft tissue retractor, and a second 5–10 mm incision in the 8th intercostal space in the posterior axillary line without a port. During the procedure, a thorough assessment of the parietal and visceral pleura, lung, and diaphragm was conducted. Any bullous lesions were excised, and diaphragmatic defects were either directly reconstructed or partially excised. The diaphragmatic reconstruction was conducted with the use of single or running unabsorbable Ticron 2 sutures with the polypropylene surgical mesh. The procedure concluded with a complete pleurectomy, and chest drain placement.

The standardized thoracolaparoscopy included simultaneous laparoscopy and videothorascopy while positioned in lateral decubitus position. This positioning precluded adequate inspection of the pelvis and limited the feasibility of extensive resection procedures within the pelvic organs. Consequently, intraoperative assessment of the pelvic cavity by gynaecologic surgeons was not performed due to the extent and complexity of the procedure. However, given the high prevalence of concomitant pelvic endometriosis, patients were referred for gynecological evaluation and management. The placement of the thoracic incisions was performed as described above, while the two 10 mm laparoscopic ports were inserted on the margins of costal arch in the middle and anterior axillary line. Abdominal insufflation was established with CO2 to an intra-abdominal pressure of 10 cmH2O. Simultaneous thoracic and abdominal visualization was achieved using separate optical systems, and the procedure was provided concurrently by thoracic and general surgical teams to identify and treat diaphragmatic lesions. During surgery, dominant diaphragmatic lesions and/or diaphragmatic perforations were visualized and ablated from the abdominal side. Meanwhile, full-thickness resection of diaphragmatic lesions, alongside wedge excision of pulmonary lesions and pleurectomy for those with a history of pneumothorax were provided. The procedure concluded with a chest drain placement.

The indication for thoracotomy as a treatment for recurrent pneumothorax was identified only until year 2017. The muscle-sparing thoracotomy was performed with the patient in the lateral decubitus position to achieve optimal exposure of the operative field. The pleura was entered through the fifth intercostal space. Following entry, a meticulous inspection of the parietal and visceral pleura, lung parenchyma, and diaphragm was performed. Bullous lesions were excised as indicated, and any diaphragmatic defects were repaired or partially resected. The procedure concluded with a pleurectomy and/or pleurodesis and insertion of an intercostal chest drain for postoperative drainage.

In this study, the previously mentioned diaphragm reconstructions were primarily provided using successive single unabsorbable sutures, over which a Prolene mesh was placed and secured. This approach ensured adequate tension and reinforced the suture line, thereby reducing the risk of secondary diaphragmatic rupture and postoperative diaphragmatic hernia. Importantly, the mesh was used solely to reinforce the suture line and did not replace any portion of the diaphragm. When clinically significant lesions were suspected on the peritoneal surface of the diaphragm, partial full-thickness resection was performed using either simple excision or excision with an Endo stapler. The diaphragm was subsequently reinforced as described above.

Tissue samples were fixed in 10% buffered formalin for 24 h, dehydrated through graded alcohols, cleared in xylene, and embedded in paraffin at 60 °C. Sections (3–5 μm) were cut, mounted on glass slides, and stained with hematoxylin and eosin. For immunohistochemistry, 4 μm tissue microarray sections on Superfrost PLUS slides were incubated overnight and stained with ERα (EP1, Dako), PR (1E2, Ventana) and CD10 (M7308, Dako) antibodies according to the manufacturer’s protocols. Two pathologists (AC, RP) evaluated staining by light microscopy (Olympus BX43 microscope) and identifying ER and PR positivity by nuclear and CD10 positivity by membranous reactions.

In our study, no formal semiquantitative scoring system (such as H-score or Allred score) was applied for the evaluation of immunohistochemical staining. The assessment was performed in a binary manner (positive vs. negative). This approach was adopted because, in the analyzed material, staining patterns were typically unequivocal. When present, immunoreactivity for ER, PR, and CD10 was generally of consistent intensity and distribution within the lesion, without significant variability that would justify the use of a graded scoring system. In most cases, the staining pattern was effectively “all-or-none,” allowing for clear classification without the need for additional semiquantitative stratification. Positivity was therefore defined as the presence of unequivocal nuclear staining for ER and PR, and membranous staining for CD10, in lesional cells, as assessed by experienced pathologists (AC, RP).

### 2.4 Ethics statement

Ethical approval was obtained from the institutional review board (KB/134–105/2026). The requirement for informed consent was waived due to the retrospective nature of the study, and all data were de-identified prior to analysis. The study was conducted in accordance with the Declaration of Helsinki.

### 2.5 Potential sources of bias

We identified and addressed several potential sources of bias. Selection bias was minimized through consecutive inclusion from a prospectively maintained institutional database, with completeness verified by applying predefined inclusion and exclusion criteria independent of outcomes. Confounding by indication was addressed by collecting baseline covariates reflecting disease severity and assessing covariate balance using standardized mean differences. Differential follow-up, loss to follow-up, and temporal bias were evaluated by comparing follow-up patterns, conducting tipping-point analyses, and stratifying analyses by era (2012–2021 vs. 2022–2025).

### 2.6 Statistical analysis

Continuous variables were reported as medians with interquartile ranges (IQR) due to non-normal distributions and compared using the Mann–Whitney U test. Categorical variables were reported as frequencies (percentages) and compared using Fisher’s exact test. Standardized Mean Differences (SMD) were calculated to assess covariate balance between exposure groups, with SMD > 0.1 indicating potential imbalance. Quantitative variables were handled based on their distribution; age was analyzed as a continuous variable after verifying linearity, and the number of previous pneumothoraces was analyzed as a continuous predictor with the single outlier (*n* = 100) excluded for exact-method stability. The study period was dichotomized into Early (2012–2021) and Late (2022–2025) eras based on institutional protocol changes, corresponding to 20 patients in the Early era and 21 in the Late era.

Missing data for baseline covariates were minimal, though postoperative hormone therapy data were missing for 17% of patients. Recurrence data were missing for 22% of patients who were lost to follow-up (9/41). We utilized complete-case analysis for regression models but assessed the potential for informative missingness by comparing baseline characteristics between patients with complete and incomplete data. Tipping point analyses were conducted to evaluate the impact of missing outcome data under various scenarios of informative censoring. Exact-method analyses were performed on patients with complete follow-up (*N* = 32).

For the outcome of intervention-requiring recurrence, exact Logistic Regression was employed to estimate Odds Ratios (OR) and 95% Confidence Intervals (CI), which is the preferred method for handling zero events in small samples. Absolute Risk Differences (RD) were calculated using the Wald method (asymptotic). For exact logistic regression models including continuous predictors (e.g., number of previous pneumothoraces), the assumption of linearity in the logit was verified using the Box-Tidwell approach (*p* > 0.05).

For the following outcomes and exploratory risk factor analysis, we prioritized exact Inference methods to address potential instability of asymptotic variance estimators in small strata. Exact Risk Ratios (RR) and 95% CIs were calculated using cohort study methods. Comparison of categorical variables was performed primarily using Fisher’s exact test. Due to the small sample size (*N* = 31 with complete follow-up) and limited number of events, multivariable adjustment was not performed to avoid overfitting. A Bonferroni correction was applied for secondary outcomes (adjusted *α* = 0.0125). Standard regression diagnostics (e.g., residual analysis) are not applicable to exact inference methods; robustness to influential observations was instead assessed using the Fragility Index and Leave-One-Out sensitivity analyses.

Prespecified subgroup analyses explored whether the effect of diaphragmatic reconstruction varied by study era, disease severity, or concurrent procedures using interaction terms. To assess result robustness, we calculated the Fragility Index and E-values to estimate the potential impact of unmeasured confounding. All statistical tests were two-sided, and a *p* value < 0.05 was considered statistically significant.

Data analysis was performed using Stata/SE version 19.5 (StataCorp LLC, College Station, TX, USA).

## 3 Results

### 3.1 Patient characteristics and follow-up

A total of 41 female patients underwent surgical management for TES. The median age was 37 years (IQR 32–40 years). Right-sided thoracic involvement was identified in 35 patients (85.4%). Video-assisted thoracoscopic surgery was performed in 37 cases (90.2%).

**Figure 1:**
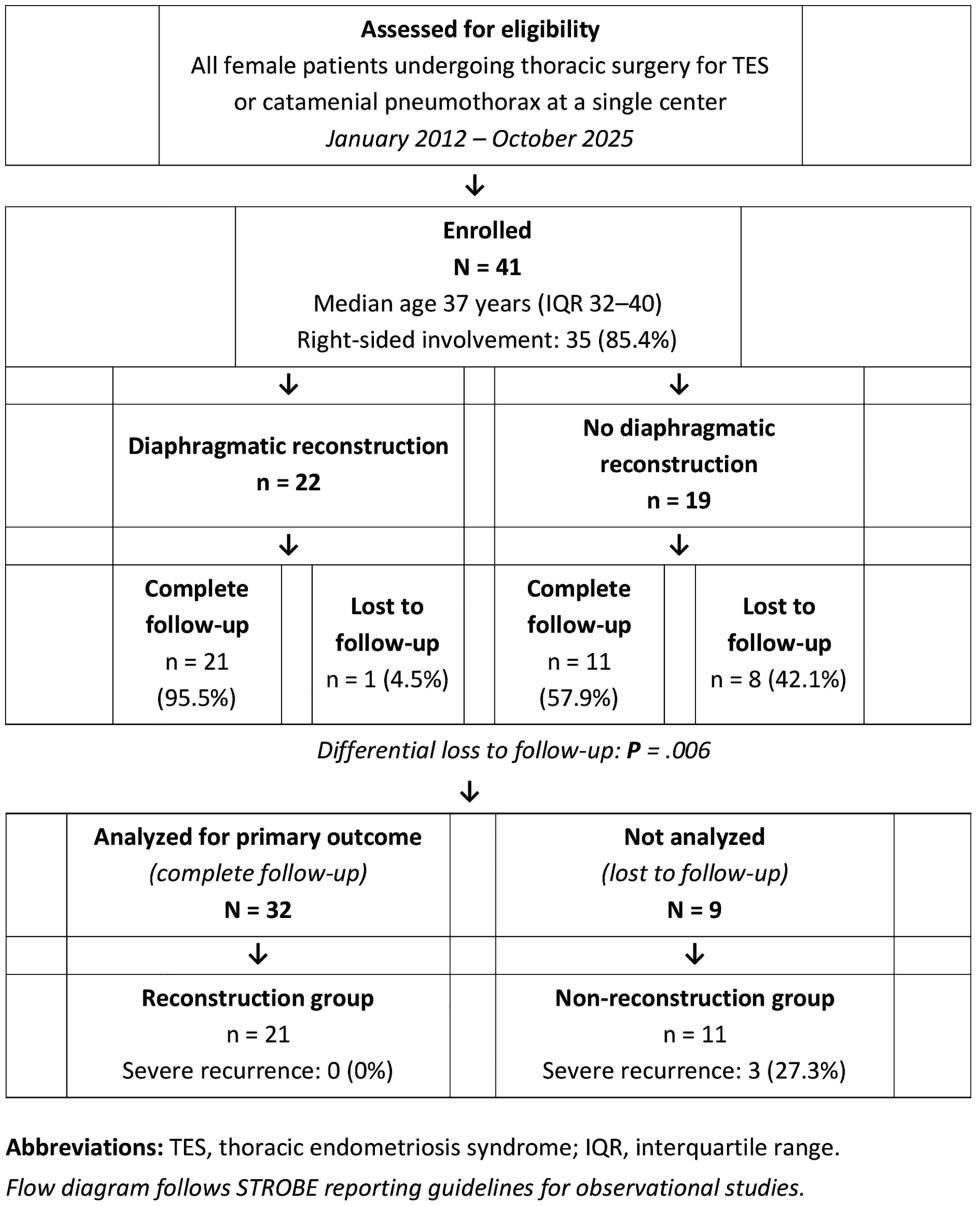
Participant flow diagram.

Of all the patients, 32 (78.0%) were included into the follow-up. Baseline characteristics, as in Table 1, were largely comparable between patients who underwent diaphragmatic reconstruction and those who did not. Differences were noted in endometriosis-related variables, with higher rates of preoperative and intraoperative diagnoses of endometriosis in the reconstruction group. Patients who underwent diaphragmatic reconstruction were potentially less likely to be lost to follow-up compared with those who did not undergo reconstruction (loss to follow-up rate 4.5% vs. 42.1%, *p* = 0.006). The participant flow is presented in Figure 1.

**Table 1:** Patients’ baseline characteristics.

Variable	Total cohort (*N* = 41)	Complete FU (*n* = 32)	LTFU (*n* = 9)	*P* value^*^	With recon (*n* = 22)	No recon (*n* = 19)	SMD^**^
Demographics							
Age, median (IQR) / mean (SD)^†^	37 (32–40)	36.7 (5.5)	34.1 (7.0)	0.320	37.5 (5.0)	34.6 (6.4)	0.492
Smoking, *n* (%)	10 (24.4%)	8 (25.0%)	2 (22.2%)	>0.999	5 (22.7%)	5 (26.3%)	−0.083
Parity (yes), *n* (%)	15 (36.6%)	14 (43.8%)	1 (11.1%)	0.119	8 (36.4%)	7 (36.8%)	−0.010
Disease characteristics							
Side (right), *n* (%)	35 (85.4%)	27 (84.4%)	8 (88.9%)	>0.999	20 (90.9%)	15 (78.9%)	0.339
Pneumothoraces, *n*, median (IQR)	4 (2–6)	4.5 (2–8)	3 (1–8)	0.066	3.5 (1–6)	4 (2–8)	0.259
Preoperative diagnosis of endometriosis, *n* (%)	22 (53.7%)	18 (56.3%)	4 (44.4%)	0.709	15 (68.2%)	7 (36.8%)	0.661
Intraoperative diagnosis of endometriosis, *n* (%)	15 (36.6%)	14 (43.8%)	1 (11.1%)	0.119	14 (63.6%)	1 (5.3%)	1.557
Surgical treatment							
VATS, *n* (%)	37 (90.2%)	28 (87.5%)	9 (100%)	>0.999	18 (81.8%)	19 (100%)	−0.667
Diaphragm reconstruction, *n* (%)	22 (53.7%)	21 (65.6%)	1 (11.1%)	0.006	—	—	—
Hormonal treatment							
Preoperative HTx, *n* (%)	13 (31.7%)	10 (31.3%)	3 (33.3%)	>0.999	7 (31.8%)	6 (31.6%)	0.005
Postoperative HTx, *n* (%)^‡^	23/34 (67.6%)	21/32 (65.6%)	2/2 (100%)	>0.999	16/21 (76.2%)	7/13 (53.8%)	0.482

^*^Fisher’s exact test or Mann–Whitney U test comparing complete follow-up vs. lost to follow-up.

^**^Standardized mean difference (SMD) comparing with reconstruction vs. no reconstruction (>0.1 indicates imbalance).

^†^Age reported as mean (SD) for both follow-up and reconstruction comparisons to enable statistical testing and SMD calculation; median (IQR) reported for total cohort.

^‡^Postoperative HTx had 7 missing values (17%); denominators shown reflect patients with available data.

Postoperative hormonal therapy was more frequently administered in the reconstruction group compared with the non-reconstruction group. In analyses comparing patients with complete follow-up to those lost to follow-up, diaphragmatic reconstruction remained more common among patients with complete follow-up, whereas comparisons of postoperative hormonal therapy were limited by missing data and small numbers in the lost to follow-up group.

No other statistically significant differences in patients’ characteristics were observed between patients with complete follow-up and those lost to follow-up.

Postoperative complications were rarely observed. In our series, two patients with diagnosed endometriosis developed postoperative diaphragmatic hernias, 11 and 26 months after prior thoracic or laparoscopic surgery, both requiring surgical repair. Among all analyzed patients, there were 2 air leaks, and 1 pleural hematoma managed with reoperation. All observed complication were classified as Clavien–Dindo grade IIIb. There were no postoperative deaths.

### 3.2 Evolution of surgical practice

**Figure 2:**
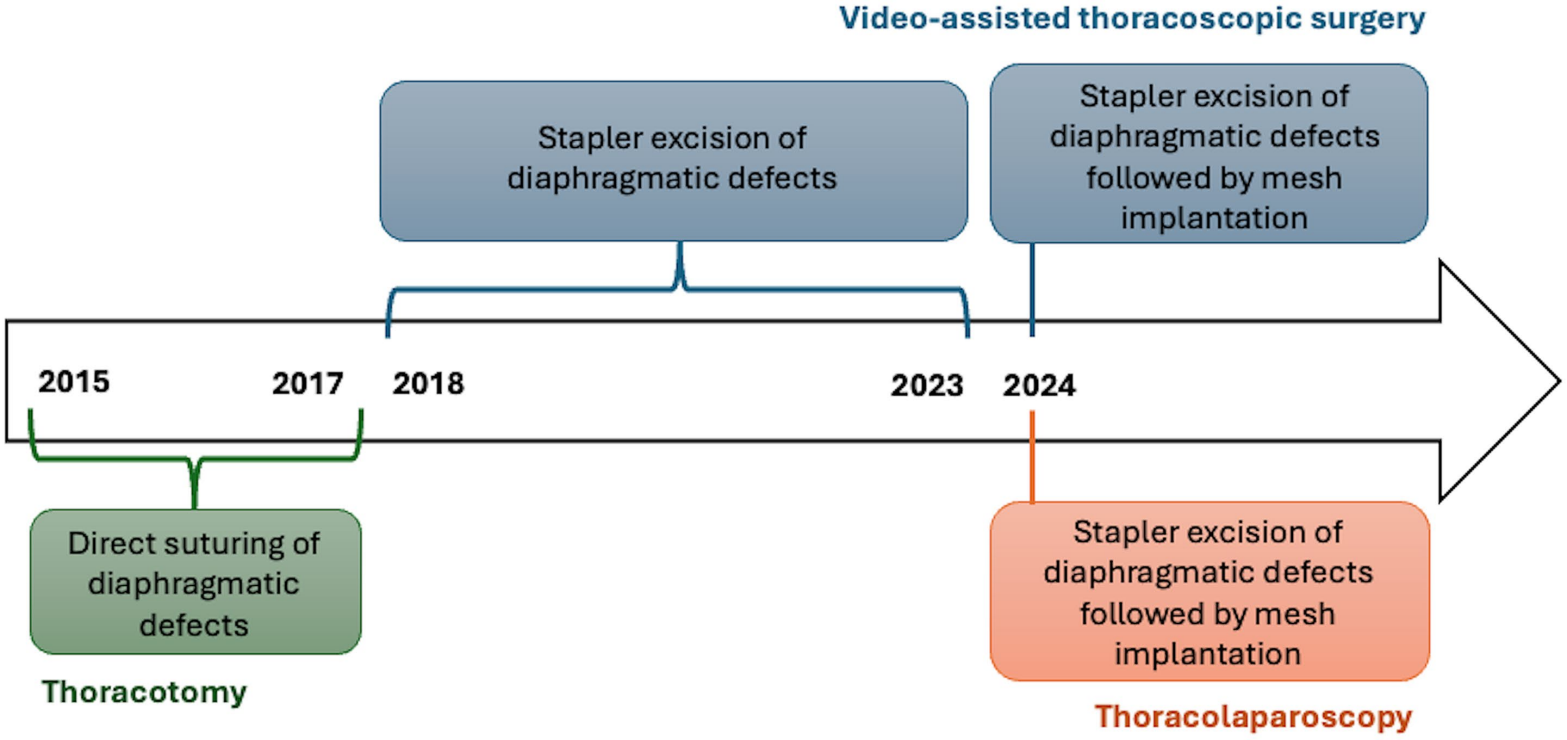
The timeline illustrating evolution of diaphragmatic intervention at the department of thoracic surgery, medical university of gdansk.

Comparison between the Early (2012–2021) and Late (2022–2025) periods, in Table 2, demonstrates significant changes in patient presentation, management and outcomes. Patients treated during the Late period were more likely to have a preoperative diagnosis of endometriosis (71.4% vs. 35.0%, *p* = 0.029) and were more frequently managed with diaphragmatic reconstruction (71.4% vs. 35.0%, *p* = 0.029). In the Early period, diaphragmatic reconstruction was associated with a not potentially beneficial reduction in the risk of any recurrence (risk ratio 0.21, 95% confidence interval 0.02–1.88; *P* = 0.164). In the Late period, the odds ratio for any recurrence was 1.80 (95% confidence interval 0.09–35.4; *p* = 0.699), though this estimate is unstable given only 2 patients without reconstruction in the Late era. The interaction between reconstruction and period was minor (*p* = 0.258), though this test was grossly underpowered to detect moderate effect modification. Intervention-requiring recurrences were numerically lower in the Late period compared with the Early period (0% vs. 18.8%), though this difference was not statistically significant (*p* = 0.226). Despite the absence of intervention-requiring recurrences in the Late period, patients continued to report postoperative pain, contributing to the composite endpoint of any recurrence. These findings are consistent with an evolution of surgical management at our department, though the small numbers preclude definitive conclusions. Figure 2 illustrates the evolution of surgical management of diaphragmatic endometriosis at our department.

**Table 2:** Comparison of surgical periods.

Variable	Early period (2012–2021) (*n* = 20)	Late period (2022–2025) (*n* = 21)	*P* value
Characteristics			
Age, mean (SD)	34.7 (6.2)	37.5 (5.3)	0.180
Preoperative diagnosis of endometriosis, *n* (%)	7 (35.0%)	15 (71.4%)	0.029
Intraoperative diagnosis of endometriosis, *n* (%)	2 (10.0%)	13 (61.9%)	0.001
Pneumothoraces, *n*, median	5	3	0.068
Management			
Diaphragm reconstruction, *n* (%)	7 (35.0%)	15 (71.4%)	0.029
Thoracotomy, *n* (%)	6 (30.0%)	0 (0%)	0.009
Outcomes (complete follow-up)	(*n* = 16)	(*n* = 16)	
Any recurrence, *n* (%)	10 (62.5%)	10 (62.5%)	>0.999
Severe recurrence, *n* (%)	3 (18.8%)	0 (0%)	0.226

### 3.3 Recurrence outcomes

Diaphragmatic reconstruction was associated with a lower rate of intervention-requiring recurrence in analyses using exact logistic regression, although the effect estimate was imprecise and the confidence interval included 1.0. Among patients with complete follow-up, no intervention-requiring recurrence was observed in the reconstruction group (0/20), compared with a 27.3% incidence in the non-reconstruction group (3/11) (Table 3). Exact logistic regression demonstrated a reduced odds of intervention-requiring recurrence associated with diaphragmatic reconstruction (OR 0.115, 95% CI: 0.00–1.152; *p* = 0.033), with consistent results observed using Fisher’s exact test (*p* = 0.033). The exact *p*-value is based on the exact score test, while the median-unbiased CI from exact logistic regression can include 1.0 in sparse data. This discordance reflects the extreme sparsity of data (zero events in one cell) and should be interpreted as indicating uncertainty about the true magnitude and direction of the association. The risk difference was −27.3 percentage points (95% confidence interval −53.6 to −1.0).

**Table 3:** Recurrence Outcomes in group after diaphragmatic reconstruction.

Outcome	No reconstruction (*n* = 11)	With reconstruction (*n* = 21)	Risk difference (95% CI)	RR / OR (95% CI)^†^	*P* value^‡^
Severe recurrence	3 (27.3%)	0 (0%)	−27.3% (−53.6, −1.0)	OR 0.115 (0.00–1.152)*	0.033
Pneumothorax recurrence	5 (45.5%)	4 (19.0%)	−26.4% (−60.3, 7.5)	RR 0.419 (0.140–1.251)	0.213
Pain recurrence	4 (36.4%)	11 (52.4%)	16.0% (−19.5, 51.6)	RR 1.440 (0.596–3.479)	0.472
Any recurrence	8 (72.7%)	12 (57.1%)	−15.6% (−49.4, 18.2)	RR 0.786 (0.468–1.319)	0.465

†Estimates derived from exact inference methods (exact logistic for severe recurrence; exact risk ratios for others) to address small sample size.

‡Primary inference from exact logistic regression for severe recurrence; Fisher’s exact test reported as supportive. Fisher’s exact test used for other outcomes.

### 3.4 Analysis of risk factors

Analyses of potential risk factors, summarized in Table 4, presents a higher preoperative pneumothorax burden associated with an increased risk of postoperative pneumothorax recurrence in exact logistic regression analysis, with an OR 1.381 per event (95% CI: 1.071–1.913; *P * = 0.005; analysis excludes the single outlier *n* = 100 for stability, *N* = 31). Postoperative HTx was not significantly associated with any recurrence outcome in univariable analysis (Table 4), though this null finding should be interpreted cautiously given the small sample size and the imbalance in postoperative HTx use between reconstruction groups (SMD = 0.482). The exact risk ratio analysis showed no statistically significant association between postoperative HTx and any recurrence outcome (Table 5). Postoperative HTx, including treatment type, was documented for all patients with complete follow-up (*n* = 32). Dienogest was the most used HTx (*n* = 15), followed by intrauterine contraception (*n* = 4) and combined oral contraception (*n* = 3), relugolix was rarely used (*n* = 1), and 9 patients received no postoperative HTx.

**Table 4:** Analysis of risk factors.

Predictor	Any recurrence (Exact RR)	Pain recurrence (Exact RR)	Pneumothorax recurrence (Exact RR/OR)	Severe recurrence (*P* value)
Age (per year)	OR 0.99 (0.86–1.13)†	OR 1.04 (0.92–1.20)†	OR 0.94 (0.80–1.08)†	0.236‡
Smoking (yes)	1.29 (0.76–2.17)	1.50 (0.73–3.07)	1.50 (0.48–4.65)	>0.999
Parity (yes)	1.29 (0.76–2.18)	1.47 (0.70–3.07)	1.61 (0.53–4.90)	0.568
Side (right)	1.05 (0.48–2.27)	0.74 (0.32–1.70)	— (Zero events)	>0.999
Pneumothoraces, *n*	OR 1.15 (0.93–1.48)†	OR 1.05 (0.87–1.28)†	OR 1.381 (1.071–1.913)†	0.526 §
Preoperative diagnosis of endometriosis	1.44 (0.80–2.62)	1.56 (0.69–3.52)	0.97 (0.32–2.96)	>0.999
Intraoperative diagnosis of endometriosis	0.86 (0.49–1.50)	1.13 (0.54–2.35)	0.64 (0.19–2.13)	0.238
Diaphragm reconstruction	0.786 (0.468–1.319)	1.440 (0.596–3.479)	0.419 (0.140–1.251)	0.033
Preoperative HTx	1.47 (0.90–2.40)	1.47 (0.72–2.99)	2.75 (0.93–8.11)	>0.999
Postoperative HTx	0.97 (0.56–1.70)	1.44 (0.60–3.48)	1.05 (0.32–3.40)	>0.999
Late period	1.00 (0.58–1.71)	1.50 (0.70–3.23)	0.50 (0.15–1.66)	0.226

†Reported as exact odds ratio due to continuous variable nature. ‡Exact logistic score test P (Fisher’s exact test does not apply to continuous predictors). § Exact rank-sum P (only 3 severe events; exact logistic unreliable). For all other rows in the Intervention-requiring Recurrence column, p values are from Fisher’s exact test. The risk factor analyses in Table 4 are exploratory and hypothesis-generating. Given the large number of comparisons (10 predictors across 4 outcomes), individual significant results should be interpreted with caution and require confirmation in independent studies.

**Table 5:** Recurrence outcomes by postoperative HTx.

Outcome	Exact RR (95% CI)	*P* value
Any recurrence	0.97 (0.56–1.70)	>0.999
Pain recurrence	1.44 (0.60–3.48)	0.472
Pneumothorax recurrence	1.05 (0.32–3.40)	>0.999
Severe recurrence (Fisher *P*)	-	>0.999

### 3.5 Histopathological confirmation

To confirm the histopathological diagnosis of TES, intraoperative specimens underwent accurate staining. Due to the limited use of immunohistochemical techniques in earlier years, patients were divided into two cohorts: those operated on before 2022, when such staining was not routinely performed, and those operated on from 2022 onward. This distinction reflects the implementation of more effective diagnostic protocols that significantly improved histopathological detection at our institution. A detailed characterization of markers expression in the newly diagnosed cases is presented in Table 6.

**Table 6:** Results of histopathological confirmation of all patients.

Staining of tissue samples	Patients, *n* = 41	
	Patients operated on until end of 2021 *n* = 20 (%)	Patients operated on since 2022 *n* = 21 (%)
Endometriosis diagnosed in histopathology staining	0 (0%)	19 (90%)
ER+	0 (0%)	12 (57%)
PR+	0 (0%)	5 (24%)
CD10+	0 (0%)	6 (29%)

### 3.6 Clinical effect size

For the primary outcome of intervention-requiring recurrence, clinical effect sizes were calculated. The Absolute Risk Reduction (ARR) was 27.3% (95% CI: 1.0 to 53.6%), with a Number Needed to Treat (NNT) of 3.7. These ARR/NNT values are descriptive (not causal), derived from observed proportions, and should be interpreted cautiously given sparse events, baseline imbalances, and differential follow-up. They should not be used to estimate future treatment effects but rather to describe the magnitude of difference observed in this specific cohort.

Higher preoperative pneumothorax count was associated with postoperative pneumothorax recurrence in exact logistic regression (OR 1.381 per event, 95% CI: 1.071–1.913; *p* = 0.005). This finding remains statistically important after Bonferroni correction for multiple secondary comparisons (adjusted *α* = 0.0125).

### 3.7 Sensitivity and robustness analysis

The Fragility Index for the primary outcome was 1 (Table 7), meaning the statistical significance would be lost if a single non-event in the reconstruction group became an event. The E-value for the exact OR point estimate was 16.91, representing the strength of association an unmeasured confounder would need with both reconstruction and severe recurrence to nullify the observed effect. However, because the upper confidence limit (1.152) crosses the null, the E-value for the confidence interval bound is approximately 1.0, indicating that even minimal unmeasured confounding could explain the observed association at the confidence interval level. In tipping point analysis, if 1 of the patients lost to follow-up in the reconstruction group had experienced a severe recurrence, the comparison would shift to 1/22 vs. 3/19 with Fisher *p* = 0.321 (loss of significance). Conversely, if all 8 non-reconstruction patients lost to follow-up were assumed to be non-events, the incidence in that group would decrease to 3/19 (15.8%), and the association with reconstruction would no longer be statistically significant (Fisher *p* = 0.092). These results highlight that the observed potential benefit is highly dependent on the assumption that outcomes in the lost-to-follow-up population follow the pattern of those observed.

**Table 7:** Sensitivity analysis summary.

Metric	Value
Fragility index	1
E-value (point estimate)	16.91
LTFU tipping point (recon)	1 event
LTFU tipping point (no recon)	0 events among lost

## 4 Discussion

This study represents a single-institution, retrospective cohort analysis of consecutive patients treated surgically for thoracic endometriosis. By evaluating a well-defined population managed within a uniform diagnostic and therapeutic framework, we aimed to clarify the clinical relevance of diaphragmatic involvement in this rare condition. Our findings demonstrate that patients presenting with catamenial pain frequently harbor endometriotic foci within the diaphragm, supporting the concept that this localization plays a central role in symptom generation. However, these results should be interpreted cautiously, as causality cannot be established and observation is likely influenced by the extent of intraoperative exploration. Importantly, meticulous intraoperative assessment allowed reliable identification of these lesions, which might otherwise remain unrecognized. We further show that surgical management incorporating targeted diaphragmatic intervention results in satisfactory therapeutic outcomes. These observations underscore the importance of a comprehensive surgical strategy when managing thoracic endometriosis, particularly in symptomatic patients.

Although four principal mechanisms have been proposed to explain the pathogenesis of catamenial pneumothorax - namely diaphragmatic defects, transdiaphragmatic passage of air, hormonally mediated pleural changes, and metastatic implantation of endometrial tissue (8, 9) - our findings suggest that not all contribute equally. In particular, involvement of the pulmonary (visceral) pleura by endometriotic implants, together with transdiaphragmatic passage of air, appears to represent the predominant pathogenic pathways (10, 11). These mechanisms provide a coherent explanation for the cyclic clinical presentation and the frequent coexistence of pleural and diaphragmatic abnormalities. Accordingly, they should be specifically considered during intraoperative assessment and surgical planning (12). Compared with existing pathogenic models, our results align most closely with studies emphasizing transdiaphragmatic air passage and pleural implantation as dominant mechanisms. However, this interpretation is based on indirect intraoperative observations rather than mechanistic proof and the alternative theories cannot be excluded and may be under-recognized.

An argument supporting the pivotal role of transdiaphragmatic air passage is the presence of a characteristic popping sensation reported by some patients. This symptom is most plausibly explained by the movement of small air bubbles from the peritoneal cavity through diaphragmatic defects into the pleural space. In our cohort, this phenomenon was observed in approximately 12% of patients with endometriosis presenting with chest-related symptoms (13). Given its distinctive and reproducible nature, we believe that this complaint reflects a specific underlying pathophysiological mechanism. Accordingly, the popping sensation may be considered a pathognomonic clinical feature of diaphragm defect-related catamenial pneumothorax. From a clinical perspective, accurate questioning about described symptom may improve preoperative exam and guide more focused intraoperative assessment.

Although our data do not allow definitive conclusions, our findings suggest that alternative mechanisms—such as local prostaglandin effects or hematogenous dissemination—probably play a comparatively minor role in the pathogenesis of TES.

Most symptoms of thoracic endometriosis syndrome manifest between 35 and 44 years of age, with a reported mean age of 36.5 years, which is consistent with the results observed in our center (9, 14).

Conformably with previous reports, endometriotic lesions in our cohort were most frequently identified on the right side of the diaphragm or involved the right pleural cavity (85.4%) (9, 15–17). This right-sided predominance has been repeatedly observed in thoracic endometriosis and catamenial pneumothorax and remains one of the characteristic features of the disease. A commonly proposed explanation relates to the physiological dynamics of peritoneal fluid, which tend to favor directional movement from the pelvic cavity toward the right subdiaphragmatic region. Such fluid circulation may facilitate the transport and implantation of endometrial cells on the right hemidiaphragm, with subsequent extension into the right thoracic cavity. This mechanism offers a plausible anatomical and physiological rationale for the marked lateralization observed in clinical practice 18, 19. Moreover, the predominance of right-sided pneumothorax supports the pivotal role of diaphragmatic defects and subsequent transdiaphragmatic spread into the right pleural cavity through full-thickness diaphragmatic involvement, whereas metastatic and prostaglandin-related theories would not be expected to demonstrate a right-sided predilection.

Thoracic endometriosis syndrome is not manifested solely by recurrent pneumothorax, reported in up to 50% of cases (16), but is more commonly dominated by severe pain, which has been described in approximately 77% of patients, often reaching high intensity levels (up to 8/10 on pain scales) (15, 16). While some studies indicate that hemoptysis is a presenting symptom in a substantial proportion of patients, with reports of up to 53% of women seeking medical attention for this complaint, our clinical experience suggests that hemoptysis is considerably less frequent. In contrast to studies reporting higher rates of hemoptysis or pleural effusion, these manifestations were uncommon in our cohort, highlighting variability across cohorts and suggesting that pain-driven presentations may be underrecognized. Bobbio reported pleural effusion or diaphragmatic hernia frequently but, in our series, diaphragmatic hernia was identified in only two patients, while no pleural effusion was observed (18).

The true incidence of diaphragmatic or thoracic endometriosis remains uncertain and likely varies depending on the clinical setting in which symptoms are first evaluated. Our estimates should therefore be interpreted as context-specific rather than representative. Patients may initially present to primary care physicians with pleuritic chest pain, to gynecologists managing known or suspected endometriosis, or ultimately to thoracic surgeons, who often represent the final step in the diagnostic pathway (19). Notably, data from gynecological centres report pleural pneumothorax in nearly half of patients with thoracic endometriosis syndrome (47%), implying that the prevalence of diaphragmatic endometriosis may be approximately twice that of clinically overt pneumothorax (17). Furthermore, it has been suggested that up to one third of women presenting with spontaneous pneumothorax may, in fact, have disease secondary to an underlying form of endometriosis (9). The reported prevalence of diaphragmatic endometriotic foci (39%) among patients treated for endometriosis-related pneumothorax (20) appears to be consistent with the rates observed in the present study for thoracic endometriosis accompanied by concomitant diaphragmatic involvement. The prevalence of concomitant diaphragmatic endometriosis among patients without symptoms attributable to diaphragmatic involvement was 0%, whereas it reached 53.7% in patients presenting with clinical features suggestive of diaphragmatic endometriosis. Overall, diaphragmatic endometriosis was identified in 39.0% of the study population. Moreover, comprehensive assessment of the diaphragm is possibly best achieved with a combined thoracolaparoscopic approach in the lateral position, which allows effective visualization and removal of most diaphragmatic lesions (21). This highlights an important clinical implication that combined thoracolaparoscopic approaches may improve diagnostic yield and may be considered in selected patients.

Some authors state that VATS represents the gold-standard (22) or reference (23) approach in the management of diaphragmatic endometriosis. Thoracolaparoscopy provides superior access to the entire diaphragm, mainly due to the possibility of complete visualization, as reported by Nezhat in 2009 and 2014; this view is also consistent with our own observations. It should be emphasized that the presence of endometriotic foci in the anterior part of the diaphragm identified during laparoscopy may indicate a higher burden of implants in its central and posterior portions (23, 24). Moreover, in Bobbio’s series the utility incision was performed to a length of approximately 7 cm, which in 2025 would in many centres be considered closer to a thoracotomy than to VATS. In our practice, recurrent cases were managed with thoracotomy until the year 2017 however, we currently believe that patients with thoracic endometriosis can be effectively treated using a VATS approach with a utility incision < 4 cm in length (15). Thoracotomy is a very rarely used approach (17, 25, 26),and is associated with worse outcomes and greater perioperative trauma (27). Smith states that the optimal approaches to diaphragmatic endometriosis are laparoscopy and laparotomy, with VATS reserved for selected cases. In contrast, our results indicate that VATS is a suitable approach to diaphragmatic endometriosis also in situations where complete resection with diaphragmatic reconstruction is required (24). Moreover, no intervention-requiring recurrences were observed from 2022 onward, indicating a trend toward a lower recurrence rate that may be associated with the evolution of surgical management of diaphragmatic endometriosis at our department. However, it should be noted that comparisons between techniques within this study may be inherently limited.

Smith proposes a classification of diaphragmatic endometriosis based on the depth of infiltration, in which the process invariably begins with superficial peritoneal involvement (grade A) and progresses to full-thickness diaphragmatic infiltration (grades D and E). Such an approach provides a rationale for broadly qualifying patients for simultaneous thoracolaparoscopy, as the extent of lesions identified during thoracoscopy alone may not accurately reflect the true degree of diaphragmatic involvement. One may, however, argue with Smith’s classification by proposing that grade E be redefined from full-thickness diaphragmatic infiltration with neural involvement to full-thickness infiltration associated with an overt pleuroperitoneal fistula. In this context, a subdivision into E1 (without fistula) and E2 (with fistula or herniation) may be considered (24).

Regarding surgical reconstruction of the diaphragm, Bobbio described the use of direct sutures with Gore-Tex when required (15). Smith, in addition to full-thickness excision of lesions classified as grades D and E, also proposes local ablative techniques for superficial disease (grades A–C), including CO₂ laser vaporization, bipolar electrocoagulation, and argon beam ablation (24). In the authors’ opinion, the most ergonomic and effective method for the management of full-thickness and deep lesions (grades C–E) is resection from the thoracic side using an EndoGIA stapler, followed by oversewing and reinforcement of the resection line with a polypropylene (Prolene) mesh. Such an approach appears to reduce the risk of postoperative diaphragmatic hernia (11, 20).

The previously described surgical approach based on performing pleurodesis in the majority of patients without diaphragmatic resection and without a meticulous search for all bullous lesions, including atypically located blebs (28), is not supported by the authors and was not applied in the present patient series; instead, our management strategy corresponds to that adopted in centres with the greatest clinical experience (4). Furthermore, the introduction of an obliterating agent into the pleural cavity - most commonly in a sprayed form - without effective closure of all communications between the pleural and peritoneal cavities, that is, pleuroperitoneal fistulas, may result in talc migration into the peritoneal cavity, which in turn may be associated with the induction of iatrogenic peritoneal adhesions (18).

In our experience, the surgical management of diaphragmatic endometriosis has progressively evolved (Figure 1). Initially, treatment was limited to direct suturing of diaphragmatic defects; this was followed by stapler excision, and subsequently by stapler excision combined with mesh implantation. Most recently, we have incorporated a combined thoracolaparoscopic approach, which enables optimal visualization of both diaphragmatic surfaces and facilitates radical, full-thickness diaphragmatic excision with mesh reconstruction. Similarly to other studies, in our cohort, we mostly provided VATS and thoracolaparoscopy approaches.

In our study, 53.7% of patients had a history of coexisting, previously diagnosed pelvic endometriosis, which is consistent with data reported by other authors. Previous studies have demonstrated that pelvic endometriosis is present in 50–86% of patients with thoracic endometriosis (28–30). In contrast, other authors have reported coexistence of pelvic endometriosis in only 20% of cases (17). We believe that, in the significant majority of patients, diaphragmatic and pleural endometriosis represent a consequence of long-standing pelvic endometriosis.

Postoperative hormonal therapy has been widely advocated as an adjunct to surgical treatment to reduce recurrence of endometriosis-related pneumothorax. Hwang reported that none of the six patients receiving postoperative hormonal therapy experienced recurrence (17). Several studies have suggested the benefit of combined surgical and postoperative hormonal treatment in reducing recurrence rates (5, 28, 31–33). Alifano et al. recommended the use of surgery combined with GnRH agonist therapy for 6–12 months, while Marshall et al. observed that, in contrast to hormonal therapies allowing menstruation, GnRH agonists effectively suppressed catamenial pneumothorax recurrence (34). In the large cohort study by Tsuboshima et al., postoperative hormonal therapy was administered in 28% of patients, most commonly dienogest, and was significantly associated with a reduced risk of postoperative recurrence in multivariable analyses (19). The authors hypothesized that hormonal therapy may control residual endometrial tissue and prevent recurrence provided that pleural endometrial lesions are resected as completely as possible. Conversely, some studies failed to demonstrate a significant benefit of postoperative hormonal therapy in reducing recurrence (35), and Subotic et al. suggested that routine use of GnRH analogues for 6–12 months should be reconsidered and further evaluated (36). In our opinion, however, evidence from large clinical series sufficiently supports the use of postoperative hormonal therapy following surgical treatment of thoracic endometriosis. Hormonal treatment should be considered with caution or temporarily withheld in patients with a high perioperative thromboembolic risk (19). We believe that postoperative hormonal therapy administered for a minimum of six months contributes to minimizing the risk of recurrent pneumothorax and therefore recommend its use in this patient population. Pathak, in a best-evidence review synthesizing data from 13 studies, demonstrated that pneumothorax recurrence was lowest when surgical management was combined with postoperative hormonal therapy, with no recurrences observed in the pooled analysis. In contrast, markedly higher recurrence rates were reported when single-modality strategies were applied, including hormonal therapy alone (59%), isolated diaphragmatic repair (33%), and surgery alone (63%), highlighting the necessity of a multimodal approach in the management of catamenial pneumothorax (37). Tsuboshima et al. reported postoperative thoracic endometriosis–related pneumothorax recurrence in 27% of 248 surgically treated patients, while postoperative hormonal therapy was administered in 28% of cases, most commonly dienogest. The authors suggested that hormonal treatment may suppress residual endometrial tissue and thereby reduce recurrence, particularly when pleural endometriotic lesions are excised as extensively as possible (19). In our cohort, the low recurrence rate of pneumothorax (25.8%) is likely attributable to the high proportion of patients with diagnosed diaphragmatic endometriosis enabling targeted surgical management, as well as to the widespread use of postoperative hormonal therapy.

Relugolix combination therapy represents a valuable addition to the current medical options for endometriosis, addressing an unmet need for effective and well-tolerated treatments suitable for long-term use (35, 36). Clinical trial data demonstrate meaningful and sustained reductions in endometriosis-associated pain, accompanied by an acceptable safety profile, which may decrease reliance on opioid analgesics and limit the need for repeated surgical interventions (38). These findings support the potential role of relugolix combination therapy in improving quality of life and facilitating long-term medical management for women with endometriosis (38). It should be emphasized that relugolix combination therapy does not currently have a specific indication for the treatment of thoracic endometriosis. Nevertheless, in patients with concomitant pelvic endometriosis who have previously undergone surgical and/or hormonal treatment, relugolix may represent a reasonable option for long-term medical management of endometriosis-related symptoms. The potential impact of relugolix on preventing recurrences of endometriosis-related pneumothorax remains unclear; however, this uncertainty itself constitutes an important rationale for future prospective studies evaluating its role in the management of thoracic endometriosis.

Haga emphasized that thoracic endometriotic lesions are often small and that endometrial glands are identified in only approximately 25% of resected specimens, making immunohistochemical analysis particularly valuable for their detection. The observation that more than half of thoracic endometrial stromal cells stained positively for smooth muscle actin, together with the presence of smooth muscle–associated thoracic endometriosis, suggests a potential capacity for smooth muscle differentiation within these lesions, although further studies are required to confirm this hypothesis (39). Kawaguchi highlighted that, although classic histopathologic features of endometriosis consist of a triad of endometrial glands, stroma, and hemosiderin-laden macrophages, endometrial glands are frequently absent in thoracic endometriosis, in contrast to pelvic disease. Diagnosis is therefore particularly challenging when only small stromal foci are present within the pulmonary parenchyma, and distinction between endometrial stroma and inflammatory cells may be difficult on hematoxylin–eosin staining alone. Given these limitations, the authors concluded that diagnostic criteria based on immunohistochemistry are required for thoracic endometriosis (40). It has been reported that thoracic endometriosis can be diagnosed in fewer than 4% of patients using hematoxylin–eosin staining alone. In our cohort, no case of thoracic endometriosis was identified solely based on routine hematoxylin–eosin examination. Consequently, in line with previous observations, we recommend routine implementation of immunohistochemical staining in female patients undergoing surgery for recurrent pneumothorax to avoid underdiagnosis of thoracic endometriosis syndrome (7). This represents a critical practice change for pathologists and surgeons to move beyond H&E staining alone to avoid the probable underdiagnosis of TES.

This study has several limitations, including its retrospective design, the lack of a uniform treatment strategy throughout the study period, and a relatively small study population, which precluded more advanced statistical analyses. The study includes diverse sources of bias, emphasising selection bias, attrition bias and temporal bias, which collectively limit interpretation. Additionally, specific data on the duration of postoperative HTx were not systematically captured in standardized form. Moreover, despite the extensive experience of the authors’ center, it cannot be excluded that some surgically treated patients in whom thoracic endometriosis or diaphragmatic endometriotic lesions were not identified, or in whom the clinical presentation did not allow a definitive diagnosis of catamenial pneumothorax, may in fact have had unrecognized thoracic endometriosis or catamenial pneumothorax. Such misclassification could have contributed to selection bias.

This single-institution retrospective analysis demonstrates that diaphragmatic endometriosis is frequently present in patients with catamenial symptoms and plays a central role in thoracic endometriosis–related clinical manifestations. Comprehensive intraoperative assessment with targeted diaphragmatic intervention, together with meticulous pathological evaluation supported by immunohistochemistry, is essential for reliable lesion detection and diagnosis, without which meaningful discussion of thoracic endometriosis syndrome is difficult. Another limitation is the inclusion of patients with previously diagnosed endometriosis and more severe TES, which introduces selection bias, as these patients likely had a higher baseline risk of pneumothorax recurrence despite treatment. Preoperative hormonal therapy showed a numerically elevated but statistically non-significant association with pneumothorax recurrence that likely reflects confounding by indication rather than a causal relationship, as patients receiving preoperative hormonal therapy may represent a population with more severe or treatment-refractory disease. Additionally, postoperative hormonal therapy was more frequently administered in the reconstruction group (76.2% vs. 53.8%, SMD = 0.482), and given the established evidence for its protective effect, may partially account for the observed association between reconstruction and lower severe recurrence.

The interpretation of the association between diaphragmatic reconstruction and recurrence must be tempered by confounding by indication and collinearity. Covariate imbalances were observed, most notably for intraoperative endometriosis diagnosis. The identical percentages for preoperative diagnosis and reconstruction in the late era (71.4%) further suggest near-collinearity, where the decision to reconstruct is inextricably linked to the preoperative suspicion of disease. Furthermore, the evolution of surgical practice means that the use of VATS is highly collinear with both the study era and the adoption of reconstructive techniques. Given these dependencies and the small sample size preventing multivariable adjustment, the unadjusted associations reported here should be viewed as descriptive rather than causal. The strong collinearity between study period, surgical approach, and reconstruction further limits the ability to disentangle these effects. Importantly, loss to follow-up was differential between groups, with 42.1% of non-reconstruction patients lost compared with only 4.5% of reconstruction patients (*p* = 0.006). The direction of resulting bias depends on the unobserved outcomes among lost patients. If patients lost from the no-reconstruction group experienced high recurrence (e.g., sought care elsewhere), the true difference would be underestimated; conversely, if they did well and thus did not return, the observed association would be overestimated. Our tipping-point analysis quantifies this sensitivity: if just one of the patients lost to follow-up in the reconstruction group had experienced a severe recurrence, Fisher’s exact P would shift from 0.033 to 0.321. On the contrary, assuming all eight lost non-reconstruction patients were non-events yields Fisher *p* = 0.092. These results confirm that the statistical significance of the primary finding is fragile and should be interpreted as hypothesis-generating. The Fragility Index for the primary outcome was 1, indicating that the statistical significance of the association between diaphragmatic reconstruction and intervention-requiring recurrence is sensitive to a single event change. These findings underscore the preliminary nature of our results and the need for larger, multi-institutional studies.

## 5 Conclusion

In this retrospective, single-institution cohort, diaphragmatic reconstruction was associated with no observed intervention-requiring recurrences among patients with complete follow-up. However, this association must be interpreted with caution due to study limitations, including a small sample size (*n* = 41) and a Fragility Index of 1. Data presented in the study demonstrate that routine IHC evaluation (ER, PR, CD10) is essential for the accurate diagnosis of TES. These findings suggest that careful diaphragmatic assessment and reconstruction may improve patient outcomes, the lack of statistical robustness necessitates further validation through multi-institutional studies. Future research should focus on establishing standardized multidisciplinary protocols to further optimize long-term results in TES management.

## Data Availability

The raw data supporting the conclusions of this article will be made available by the authors, without undue reservation.
