# Supplementary material for: Beyond the pelvis: diagnostic and surgical challenges of thoracic endometriosis syndrome - a retrospective cohort study of 41 patients
Source: Front Med (Lausanne). 2026 May 28;13:1835516. doi: 10.3389/fmed.2026.1835516 (PMC13254090; doi:10.3389/fmed.2026.1835516)
Supplement: Supplementary file 1 [file Table_1.docx]

### Supplementary Table. STROBE Statement — Checklist of Items for Cohort Studies

| **Item No** | **STROBE Item** | **Reported** | **Location in manuscript** |
| --- | --- | --- | --- |
|  | **TITLE AND ABSTRACT** |  |  |
| 1a | Indicate the study’s design with a commonly used term in the title or the abstract | Yes | Title: “Retrospective Study” [recommend changing to “Retrospective Cohort Study”]; Abstract: “retrospective cohort study” |
| 1b | Provide in the abstract an informative and balanced summary of what was done and what was found | Yes | Abstract (new section added before Introduction) |
|  | **INTRODUCTION** |  |  |
| 2 | Explain the scientific background and rationale for the investigation being reported | Yes | Introduction, paragraphs 1–3 (Lines 49–53) |
| 3 | State specific objectives, including any prespecified hypotheses | Partially | Introduction, paragraph 3 (Line 53). Recommendation: state explicit primary objective with exposure, comparator, outcome, and population |
|  | **METHODS** |  |  |
| 4 | Present key elements of study design early in the paper | Yes | Materials & Methods, “Study Design and Setting” (Line 59) |
| 5 | Describe the setting, locations, and relevant dates, including periods of recruitment, exposure, follow-up, and data collection | Yes | Materials & Methods (Lines 59, 63, 65) |
| 6a | Give the eligibility criteria, and the sources and methods of selection of participants. Describe methods of follow-up | Yes | “Participants” (Lines 61–65); follow-up methods described |
| 6b | For matched studies, give matching criteria and number of exposed and unexposed | N/A | Not a matched study. Exposure groups described in “Data Collection and Definitions” (Line 69) |
| 7 | Clearly define all outcomes, exposures, predictors, potential confounders, and effect modifiers. Give diagnostic criteria, if applicable | Partially | Outcomes defined in “Data Collection and Definitions” and “Statistical analysis.” Recommendation: add formal outcome definitions for severe recurrence, pneumothorax recurrence, pain recurrence, and any recurrence in Methods |
| 8 | For each variable of interest, give sources of data and details of methods of assessment (measurement). Describe comparability of assessment methods if there is more than one group | Yes | “Data Collection and Definitions” (Line 67–69); histopathology methods (Line 77) |
| 9 | Describe any efforts to address potential sources of bias | Yes | “Potential sources of bias” (Lines 83–85); SMD calculation; tipping-point analysis |
| 10 | Explain how the study size was arrived at | Partially | Not explicitly stated in current manuscript. Recommendation: add “The sample size was determined by consecutive enrollment of all eligible patients (convenience sample). No formal a priori power calculation was performed.” |
| 11 | Explain how quantitative variables were handled in the analyses. If applicable, describe which groupings were chosen and why | Yes | “Statistical analysis” (Line 89): age as continuous, pneumothoraces as continuous with outlier exclusion, era dichotomized |
| 12a | Describe all statistical methods, including those used to control for confounding | Yes | “Statistical analysis” (Lines 87–99): exact logistic regression, Fisher’s exact test, SMD, Bonferroni correction |
| 12b | Describe any methods used to examine subgroups and interactions | Yes | Line 97: “Prespecified subgroup analyses explored whether the effect of diaphragmatic reconstruction varied by study era…” |
| 12c | Explain how missing data were addressed | Yes | Line 91: complete-case analysis with tipping-point sensitivity analysis |
| 12d | If applicable, explain how loss to follow-up was addressed | Yes | Lines 85, 91: tipping-point analyses; comparison of baseline characteristics between FU and LTFU groups |
| 12e | Describe any sensitivity analyses | Yes | Line 97: Fragility Index, E-values; new Sensitivity Analysis section in Results |
|  | **RESULTS** |  |  |
| 13a | Report numbers of individuals at each stage of study — e.g., numbers potentially eligible, examined for eligibility, confirmed eligible, included in the study, completing follow-up, and analysed | Yes | Figure 2 (flow diagram, new); Results narrative (Line 107); Table 1 |
| 13b | Give reasons for non-participation at each stage | Partially | LTFU described; reasons for non-participation not detailed. Recommendation: add reasons for LTFU if available |
| 13c | Consider use of a flow diagram | Yes | Figure 2 (new flow diagram added) |
| 14a | Give characteristics of study participants (e.g., demographic, clinical, social) and information on exposures and potential confounders | Yes | Table 1 (baseline characteristics with SMD for covariate balance) |
| 14b | Indicate number of participants with missing data for each variable of interest | Partially | Table 1 footnote ‡ for postoperative HTx (17% missing). Recommendation: explicitly report missing data counts for all variables |
| 14c | Summarise follow-up time (e.g., average and total amount) | Yes | Line 65: “Follow-up duration ranged from 3 to 156 months (median 36 months)” |
| 15 | Report numbers of outcome events or summary measures over time | Yes | Tables 2, 3; Results narrative (Lines 145, 167) |
| 16a | Give unadjusted estimates and, if applicable, confounder-adjusted estimates and their precision (e.g., 95% confidence interval). Make clear which confounders were adjusted for and why they were included | Yes | Table 3 (unadjusted exact OR/RR with 95% CI); Table 4 (univariable risk factors). No multivariable adjustment performed (justified by EPV constraints, Line 95) |
| 16b | Report category boundaries when continuous variables were categorized | Yes | Era split: Early (2012–2021) vs Late (2022–2025) defined in Methods (Line 89) |
| 16c | If relevant, consider translating estimates of relative risk into absolute risk for a meaningful time period | Yes | Table 3: Risk Differences reported alongside RR/OR. Clinical Effect Size section: ARR 27.3%, NNT 3.7 |
| 17 | Report other analyses done — e.g., analyses of subgroups and interactions, and sensitivity analyses | Yes | Table 2 (era-stratified); era-specific reconstruction effect; Fragility Index; E-value; tipping-point analysis (new Sensitivity Analysis section) |
|  | **DISCUSSION** |  |  |
| 18 | Summarise key results with reference to study objectives | Yes | Discussion, opening paragraphs (Lines 219–221) |
| 19 | Discuss limitations of the study, taking into account sources of potential bias or imprecision. Discuss both direction and magnitude of any potential bias | Partially | Line 255 (current limitations). Recommendation: expand with Fragility Index, differential LTFU, and confounding by indication discussion (see B4, B5) |
| 20 | Give a cautious overall interpretation of results considering objectives, limitations, multiplicity of analyses, results from similar studies, and other relevant evidence | Yes | Discussion (Lines 219–258); Conclusions (Line 261). Recommendation: soften language from “complete elimination” to “absence of” |
| 21 | Discuss the generalizability (external validity) of the study results | Partially | Implicit through comparison with other centers. Recommendation: add explicit statement about generalizability to other settings |
| 22 | Give the source of funding and the role of the funders for the present study and, if applicable, for the original study on which the present article is based | Yes | Funding and Conflicts of Interest statements |
